# Necroptotic Cell Death Promotes Adaptive Immunity Against Colonizing Pneumococci

**DOI:** 10.3389/fimmu.2019.00615

**Published:** 2019-04-04

**Authors:** Ashleigh Nichole Riegler, Terry Brissac, Norberto Gonzalez-Juarbe, Carlos J. Orihuela

**Affiliations:** Department of Microbiology, The University of Alabama at Birmingham, Birmingham, AL, United States

**Keywords:** *Streptococcus pneumoniae*, cell death, necrosis, necroptosis, pore-forming toxin (PFT), pneumolysin (PLY), innate and adaptive immune response, colonization

## Abstract

Pore-forming toxin (PFT) induced necroptosis exacerbates pulmonary injury during bacterial pneumonia. However, its role during asymptomatic nasopharyngeal colonization and toward the development of protective immunity was unknown. Using a mouse model of *Streptococcus pneumoniae* (*Spn*) asymptomatic colonization, we determined that nasopharyngeal epithelial cells (nEC) died of pneumolysin (Ply)-dependent necroptosis. Mice deficient in MLKL, the necroptosis effector, or challenged with Ply-deficient *Spn* showed less nEC sloughing, increased neutrophil infiltration, and altered IL-1α, IL-33, CXCL2, IL-17, and IL-6 levels in nasal lavage fluid (NALF). Activated MLKL correlated with increased presence of CD11c^+^ antigen presenting cells in *Spn*-associated submucosa. Colonized MLKL KO mice and wildtype mice colonized with Ply-deficient *Spn* produced less antibody against the bacterial surface protein PspA, were delayed in bacterial clearance, and were more susceptible to a lethal secondary *Spn* challenge. We conclude that PFT-induced necroptosis is instrumental in the natural development of protective immunity against opportunistic PFT-producing bacterial pathogens.

## Introduction

Regulated cell death without loss of membrane integrity, such as apoptosis, is a vital aspect of organ development, immunity, physiological maintenance, and wound healing (1, 2). In contrast, necrotic cell death, where organelle and cell membrane integrity are lost and cytoplasmic components are released, is generally considered to be detrimental, an unintended consequence of extreme physiological perturbation, irreversible mechanical damage, and/or catastrophic energy depletion. Yet, we now know that death by necrosis, without simultaneous activation of the pyroptotic inflammasome, is in some instances regulated by the cell (3–5). The latter suggests that a loss of cell integrity can be in some instances beneficial to the organism as a whole.

One form of programmed necrosis, called necroptosis, is canonically activated as result of death receptor ligation, e.g., tumor necrosis factor receptor 1, with concurrent caspase-8 inhibition (6, 7). This results in the activation of receptor-interacting serine/threonine protein-kinases (RIPK)-1 and RIPK-3, which then together activate mixed lineage kinase domain pseudokinase (MLKL) by phosphorylation. Activated MLKL (pMLKL) targets phosphatidylserine residues in cellular membranes leading to their dissolution (8). Importantly, cytosolic contents released from necroptotic cells serve as alarmins and signal to nearby cells of imminent danger or injury and trigger inflammation (9–11). Necroptosis is known to occur following sterile injury, such as ischemia-reperfusion (12, 13), during viral infection in cells that have blocked apoptosis (14), and during infection with bacteria that produce pore-forming toxins (PFT). In the latter circumstance, PFT-induced membrane damage results in ion dysregulation and energy depletion, which activates RIPK1 in non-canonical fashion (15–19). During pneumonia, bacterial PFTs induce necroptosis of alveolar macrophages and lung epithelial cells, exacerbate tissue injury, and contribute to reduced lung function. Common opportunistic respiratory pathogens including *Streptococcus pneumoniae (Spn), Staphylococcus aureus*, and *Serratia marcescens* have been shown to induce PFT-mediated necroptosis of lung cells; with necroptosis deficiency or its inhibition reducing injury and improving survival (15, 18).

*Spn* is the leading cause of community-acquired pneumonia and is responsible for ~1.4 million deaths annually (20, 21). For *Spn*, colonization of the nasopharynx is a prerequisite to the development of pneumonia and invasive disease (22). Within the nasopharynx, *Spn* forms biofilms (23, 24), a growth phenotype that produces and releases more of its PFT, pneumolysin (Ply) (25). Natural immunity against *Spn* typically develops as result of repeated colonization events by different serotypes during early childhood. Broad protective immunity is achieved as result of obtaining a threshold of immune recognition against conserved proteins found on the bacterial surface (26). This is in contrast to the protective antibody that is elicited by the current pneumococcal vaccines; which are composed of an unrelated protein conjugated to as many as 13 distinct *Spn* capsular polysaccharides (27–30). Thus, vaccine-immunized individuals remain susceptible to the >80 non-vaccine serotypes of *Spn* unless they have developed the aforementioned broad protective immunity that arises as result of colonization. Taken together, the global health impact of *Spn* is tremendous and *Spn* serves as an excellent prototype to examine the effects of PFT-induced necroptosis on the innate and adaptive immune response in the airway.

Herein we explored the role of PFTs in the host-pathogen interactions which occur during *Spn* colonization and serve to prevent the development of disease. Our goals were to identify whether necroptosis was initiated by Ply during asymptomatic colonization, to characterize the influence of this form of cell death on the immune responses during primary infection, and to assess the role of PFT-induced necroptosis on the generation of broad protective immunity against bacterial antigens. Importantly, our findings provide meaningful insight into the host-pathogen interactions of nasopharyngeal colonizing, PFT-producing bacteria and indicate that cell death by necroptosis is a critical driver of host immunity.

## Materials and Methods

### Mice

Animal experiments were carried out using male and female 6–12-week-old adult mice. Wildtype C57BL/6 were supplied from Jackson Labs (Sacramento, California) and MLKL^−/−^ mice in the C57BL/6 background were obtained from Dr. Warren Alexander (31) and housed in the University of Alabama at Birmingham Animal Facilities. To achieve colonization, nasal aspiration was performed on each mouse as previously described with an inoculum of ~1 × 10^5^ CFU in 10 μL saline (24). To model pneumonia, oropharyngeal aspiration with an inoculum of ~1 × 10^6^ CFU in 100 μL saline was used as previously described (18). Mice were monitored and assigned a body condition score (BCS) as detailed in Figure S1, a method adapted from Current Protocols in Mouse Biology (32). Tissue samples were collected from mice at the indicated time points after inoculation. Nasal lavage (NALF) and collection of nasal turbinates, including nasal associated lymphoid tissue (NALT) were performed post-mortem as previously described (24).

### Bacterial Strains and Culture

*S. pneumoniae* serotype 4 strain TIGR4 and serotype 2 strain D39 are previously described (33, 34). The strain carrying an isogenic deletion of pneumolysin (TIGR4Δ*ply*) and its derivative carrying a functional pneumolysin toxoid (TIGR4w433F) have also been described and validated using complemented strains (35, 36). All strains were grown on tryptic soy agar supplemented with sheep's blood (Remel R01202) overnight or in tryptic soy broth supplemented with 0.5% yeast extract (THY) at 37°C in 5% CO_2._ Bacteria in log phase growth (OD_620_ ~0.5) were used for experiments.

*Escherichia coli* expressing recombinant Ply or PspA was grown in Lysogeny Broth (LB) supplemented with kanamycin (50 μg/mL) or ampicillin (100 μg/mL), to log phase (OD_621_ ~0.3–0.5) and the HIS tagged recombinant proteins were purified by nickel column chromatography as previously described (37, 38). Recombinant protein identity was confirmed by Western. Recombinant pneumolysin activity was validated using a red blood cell hemolysis assay (39).

### Tissue Staining for Microscopy

For staining and microscopy, euthanized mice were decapitated and their heads placed in PBS with 1X protease phosphatase inhibitor cocktail on ice. Following removal of the exterior skin and flesh, dissected heads were decalcified for 4 h using RDO Decalcification solution (Election Microscopy Sciences; 3:1 in water), neutralized following manufacturer's protocol, and embedded in OCT. Sectioned samples were prepared for staining by treating in acetone for 10 min at −20°C then 70% ethanol for 5 min at −20°C. For immunofluorescence, following rehydration with PBS and permeabilization with PBS Triton-X (0.02%), samples were blocked with 5% BSA for 45 min at room temperature then probed for specified protein (1/1,000) at 4°C overnight. Samples were washed 3 times for 5 min in PBS-T (PBS-0.05% Tween20). Samples were then incubated in corresponding fluorescent secondary antibody solution for 1 h at room temperature. Blocking, probing, and detection were then repeated for additional, specified antigens. Following final detection and wash, nuclei were detected using NucBlue (Invitrogen) per manufacturer's instruction and mounted in Fluorsave (EMD Millipore). Antibody details available in Table S1. For gross histology, sections were stained by Alcian blue/ PAS according to the IHCWORLD protocol adapted and translated from the Journal of Histochemistry and Cytochemistry (40).

### Microscopy and Image Analysis

Images were captured using a Leica LMD6 with DFC3000G-1.3-megapixel monochrome camera or DFC450C-5-megapixel RGB CCD (Leica Biosystems, Buffalo Grove, IL). Exposure levels optimized on controls and maintained throughout. Wherever noted, tile scanned images were compiled using the Tile Scan stitching feature of the Leica Application Suite X (LAS X) (Leica Biosystems, Buffalo Grove, IL). Magnifications noted within figure legends along with scale bars. Mean Fluorescent Intensity (MFI) calculated using ImageJ 1.51h (National Institutes of Health, USA).

### Western Blot Analysis

Tissue homogenates in PBS were desalted using Amicon 10 kDa spin columns (Millipore). Desalted homogenates were incubated for 30 min at 4°C in Protein lysis Buffer (50 mM TrisHCl-150 mM NaCl-1% Triton X100-1X HALT Protease Phosphatase Inhibitor Cocktail), centrifuged (14,000 rpm at 4°C) for 15 min, and concentrations of the resulting the resulting whole protein lysates were determined from the supernatant using a Bicinchoninic Acid assay kit (Sigma-Aldrich) according to the manufacturer instructions. 10 μg of total protein were loaded and separated on a 10% polyacrylamide gel (Biorad) before transfer on nitrocellulose membrane (Biorad). Membranes were blocked in 5% Non-dry fat milk and washed 3 times for 5 min in TBS-0.1%Tween20 (TBST). Membranes were incubated with anti-MLKL (1/1,000), anti-pMLKL (1/1,000), anti-caspase3/Cleaved caspase3 (1/2,000) or anti-actin (1/10,000), in 5%BSA overnight at 4°C with gentle agitation. Membranes were then washed 3 times for 10 min in TBST and incubated with HRP-conjugated goat anti-rabbit (1/10,000, Jackson). Membranes were washed 3 times for 10 min in TBST, once for 5 min with TBS and signal was detected using Clarity™ Western ECL and ChemiDoc XRS+ (Biorad). Protein expression was determined by densitometry using ImageJ. Antibody details available in Table S1.

### Inhibitors, Antibodies, and Other Chemicals

The MLKL inhibitor necrosulfonamide (NSA) was obtained from Tocris Bioscience (QL, United Kingdom). To inhibit caspases, the general caspase inhibitor Z-VAD-fmk was obtained from R&D Systems (Minneapolis, MN). The lipid oxidase inhibitor Liproxstatin-1, used to inhibit Ferroptosis, was obtained from Sigma (St Louis, MO). All inhibitor concentrations listed in figure legends. Cytospins were stained with PROTOCOL® HEMA 3® Stain set from Fisher Scientific (Kalamazoo, MI) according to the manufacturer's instruction. Antibody and chemical details provided in Table S1.

### Cell Culture

FaDu (HTB-43) human pharyngeal epithelial cells were obtained from the American Type Culture Collection (Manassas, VA) and cultured in Gibco™ Minimum Essential Medium supplemented with 10% Fetal Bovine Serum (Atlanta Biologicals) and 1% Gibco™ Antibiotic-Antimycotic. Cultures were grown at 37°C in 5% CO_2_. Cell infection experiments were carried out in 96-well-plates seeded at 1 × 10^4^ cells/well and infected 24–30 h after seeding with *S. pneumoniae* at an MOI of 10 overnight (15 h). For positive lysis controls, LDH Lysis Buffer from the Pierce LDH Cytotoxicity Assay Kit was added according to manufacturer's instructions to lysis control wells 20–30 min prior to sample collection.

### Statistics

Statistical comparisons were calculated using GraphPad Prism 8 (La Jolla, CA). Comparisons between two cohorts at a single time point are calculated by Mann-Whitney *U*-test. Comparisons between groups of >2 cohorts or groups given multiple treatments were calculated by ANOVA with Tukey's (one-way) or Sidak's (two-way) *post-test* or by Kruskal-Wallis H test with Dunn's multiple comparison *post-test*, as determined by the normality of data groups. Repeated measures are accounted for whenever applicable. In all instances, data are plotted as mean ± SEM.

### Study Approval

The aim of this study was to characterize the role of necroptosis in the innate and adaptive immune responses to colonizing *Streptococcus pneumonaie*. All animal studies were performed in compliance with the federal regulations set forth in the Animal Welfare Act, the recommendations in the National Institutes of Health Guide for the Care and Use of Laboratory Animals, and the University of Alabama at Birmingham Institutional Animal Use and Care Committee (IACUC). All protocols used in this study were approved by the IACUC at the University of Alabama at Birmingham (protocols #20479 and #21231). Power calculations from past studies were used to calculate the number of mice needed to ensure statistical power. Unless otherwise noted, all *in vitro* experiments are composed of a minimum of 3 biological replicates, with ≥3 technical replicates each. All *in vivo* experiments were done with a minimum of 3 biological replicates. All results were confirmed with a minimum of two independent experiments.

## Results

### Cell Death Occurs During Asymptomatic Colonization by *S. pneumoniae*

**Figure 1:**
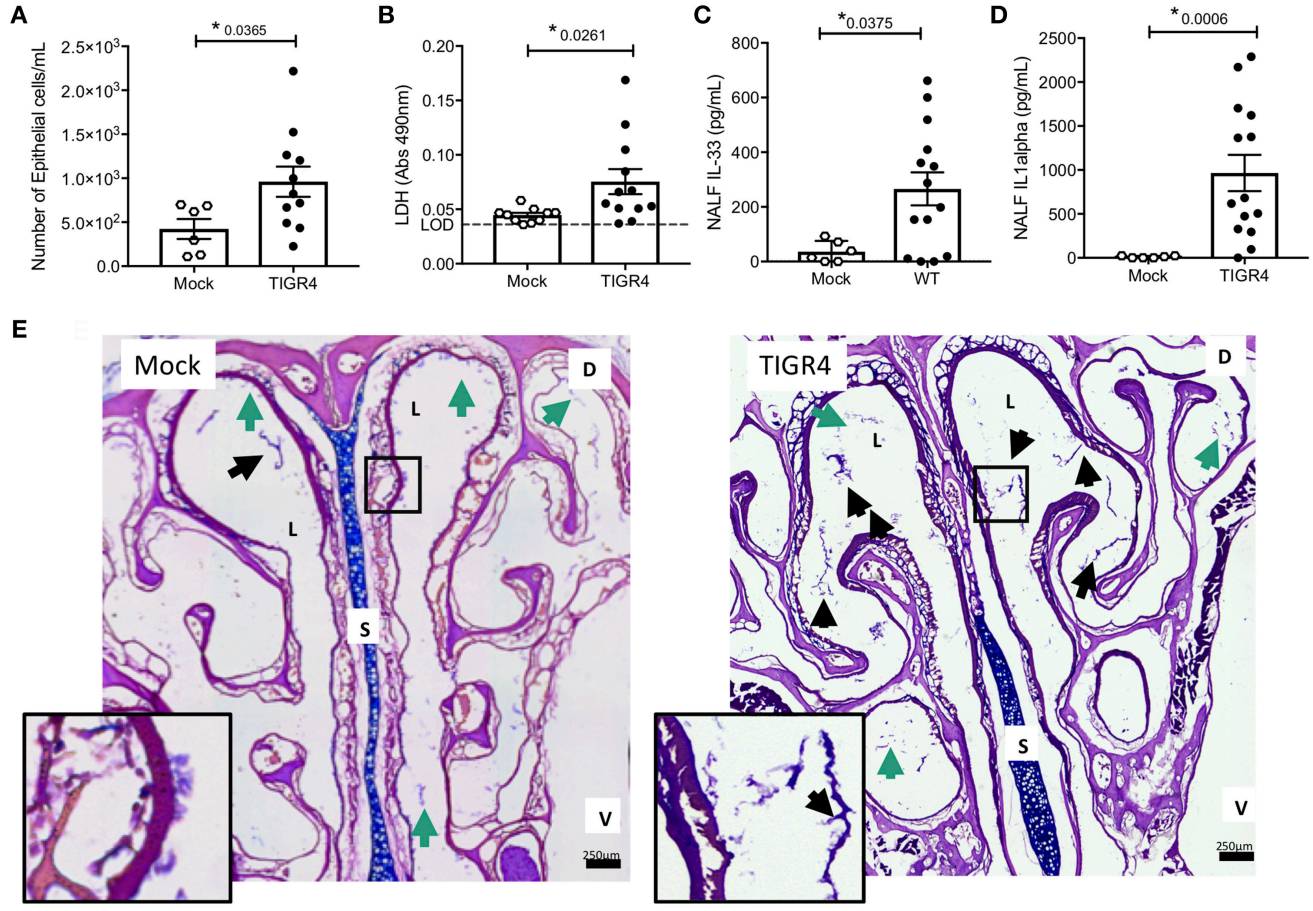
Cell damage occurs during asymptomatic colonization by *Spn*. Nasal lavage collected from wildtype C57BL/6 (WT) mice at day 7 post-inoculation with TIGR4 or PBS (Mock) analyzed for **(A)** nEC sloughing, quantified from HEMA stained Cytospin samples, **(B)** lactate dehydrogenase (LDH), dashed line indicating limit of detection, **(C)** IL-33, and **(D)** IL-1α. Mean ± SEM. Mann-Whitney *U*-test used for comparisons (*n* = 10 – 14). *P*-values listed on graph; * indicates a value < 0.05. **(E)** Representative nasal section and zoomed inset from WT mouse either mock colonized or colonized with TIGR4, collected at day 7 post-inoculation and stained with Alcian/PAS. Lumen (L), septum (S), dorsal side (D), and ventral side (V) denoted. Arrows indicating luminal clusters of epithelial cells (black) and mucus (green). Inset source region denoted by black box outline. Images Tile Scan assembled at 10x magnification. (See also Table S2 for other cytokines tested but either unchanged or below limit of detection).

Nasopharyngeal colonization was established in 6–8 week-old male and female mice by instillation of ~10^5^ CFU of serotype 4 strain TIGR4 into the nares. This resulted in a colonization burden of ~2 × 10^5^ CFU/g nasoturbinate at 7-days post-inoculation and clearance of *Spn* by 21-days post-inoculation (Figure S1A). Colonized mice appeared normal with no overt physical signs of distress (e.g., changes in gait, grooming, posture, appetite, activity, etc. Figures S1B,C). Despite this, nasal lavage fluid (NALF) collected 7-days post-inoculation, a time point associated with the initiation of adaptive immunity (41) and prior to clearance, showed greater nasopharyngeal epithelial cell (nEC) sloughing in colonized mice vs. negative controls (Figure 1A). This corresponded with increased amounts of the cell damage markers lactate dehydrogenase (LDH; Figure 1B), interleukin (IL)-33 (Figure 1C), and IL-1α (Figure 1D) in isolated NALF. Notably, IL-1α and IL-33 are pro-inflammatory cytokines released when cells die of necrosis (42, 43). Of note, we did not detect mature IL-1β in NALF from colonized or uncolonized mice (*n* = 6 mice per group, limit of detection = 31.3 pg/ml), nor were meaningful differences observed for CXCL1, IL-7, IL-12, IL-13, IFNγ, and TNFα (Table S2). Consistent with previous publications (36, 44), microscopic examination of nasal sections revealed pathological evidence of damage and inflammation in colonized mice vs. controls, including more frequent clusters of mucus and sloughed nECs in the lumen (Figure 1E); the latter being the most likely source of those detected in NALF.

**Figure 2:**
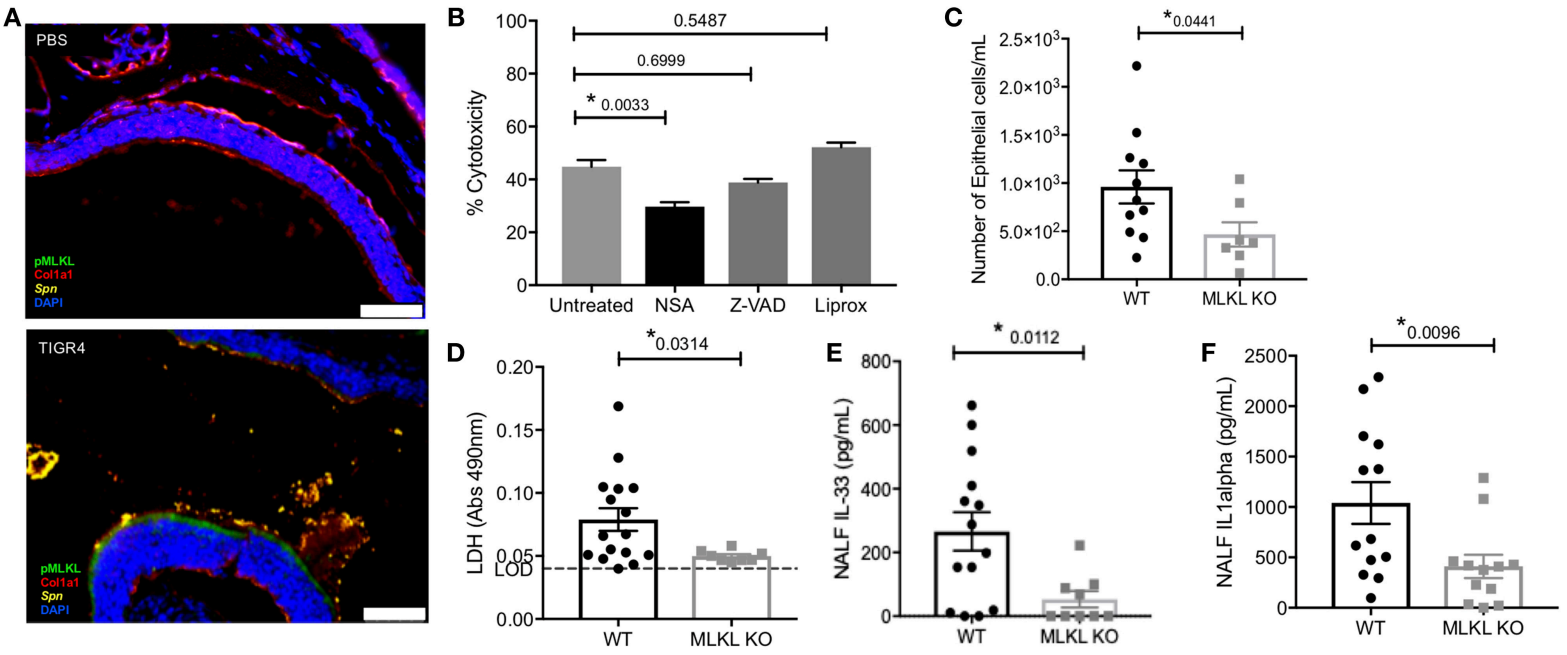
Colonizing *Spn* cause necroptosis of nEC. **(A)** Representative IF images of nasal turbinates from WT mice collected at day 7 post-inoculation with TIGR4 or PBS stained for pMLKL (green), collagen-1a (red), Spn (yellow), and DAPI (Blue). Imaged at 40X Tile Scan. Scale bars indicate 50 μm. **(B)** FaDu cell death (%cytotoxicity) as measured by LDH release from cells pre-treated with normal media or media containing 101 iM of either the MLKL inhibitor Necrosulfonamide (NSA), the general caspase inhibitor ZVAD, or the ferroptosis inhibitor Liproxstatin-1 (Liprox), for 1 h then challenged overnight (15 h ) with TIGR4 at an MOI of 10. One-way analysis of variance used for comparisons (Cytotoxicity *F* = 14.78, *p* < 0.0001, Treatment *F* = 34.38, *p* < 0.0001) (See also Figure S3A). **(C–F)** NALF from WT and MLKL KO mice colonized with TIGR4 analyzed for **(C)** nEC sloughing, **(D)** LDH, dashed line indicates limit of detection, **(E)** IL-33, and **(F)** IL-1α. LDH Absorbance at 490 nm LOD normalized to absorbance of uninfected control NALF. Mean ± SEM; Mann-Whitney *U*-test for comparisons (*n* = 10–14 animals per genotype). *P*-values listed on graph; * indicates a value < 0.05. (See also Figures S2,S3 and Table S2).

We examined the specific type of cell death that occurred during colonization, namely whether necroptosis was occurring. Nasal sections (Figure 2A) from colonized mice were positive for pMLKL, suggesting nECs were dying of necroptosis. Moreover, the same samples showed negligible amounts of cleaved caspase-3 (Figure S2). Further implicating necroptosis as the principal mode of nasopharyngeal epithelial cell death following *Spn* exposure, death of FaDu human pharyngeal cells infected with TIGR4 *in vitro* was reduced when cells were pre-treated with the necroptosis inhibitor necrosulfonamide (NSA). No protection against death was observed when FaDu cells had been pre-treated with the pan-caspase inhibitor Z-VAD-fmk (Z-VAD) or the ferroptosis inhibitor Liproxstatin-1 (Liprox) (Figure 2B). We next tested necroptosis deficient (MLKL KO) mice. Colonized MLKL KO mice showed decreased nEC sloughing (Figure 2C) as well as decreased LDH, IL-33, and IL-1α levels in NALF (Figures 2D–F) levels in NALF (Figures 2D,E), when compared to colonized WT controls. We thereby conclude that necroptosis occurred within the nasopharynx during asymptomatic colonization with *Spn*, and the inability to activate this cell death mechanism resulted in lower amounts of alarmins released by dying cells.

### Necroptosis During *Spn* Colonization Is PFT-Dependent

**Figure 3:**
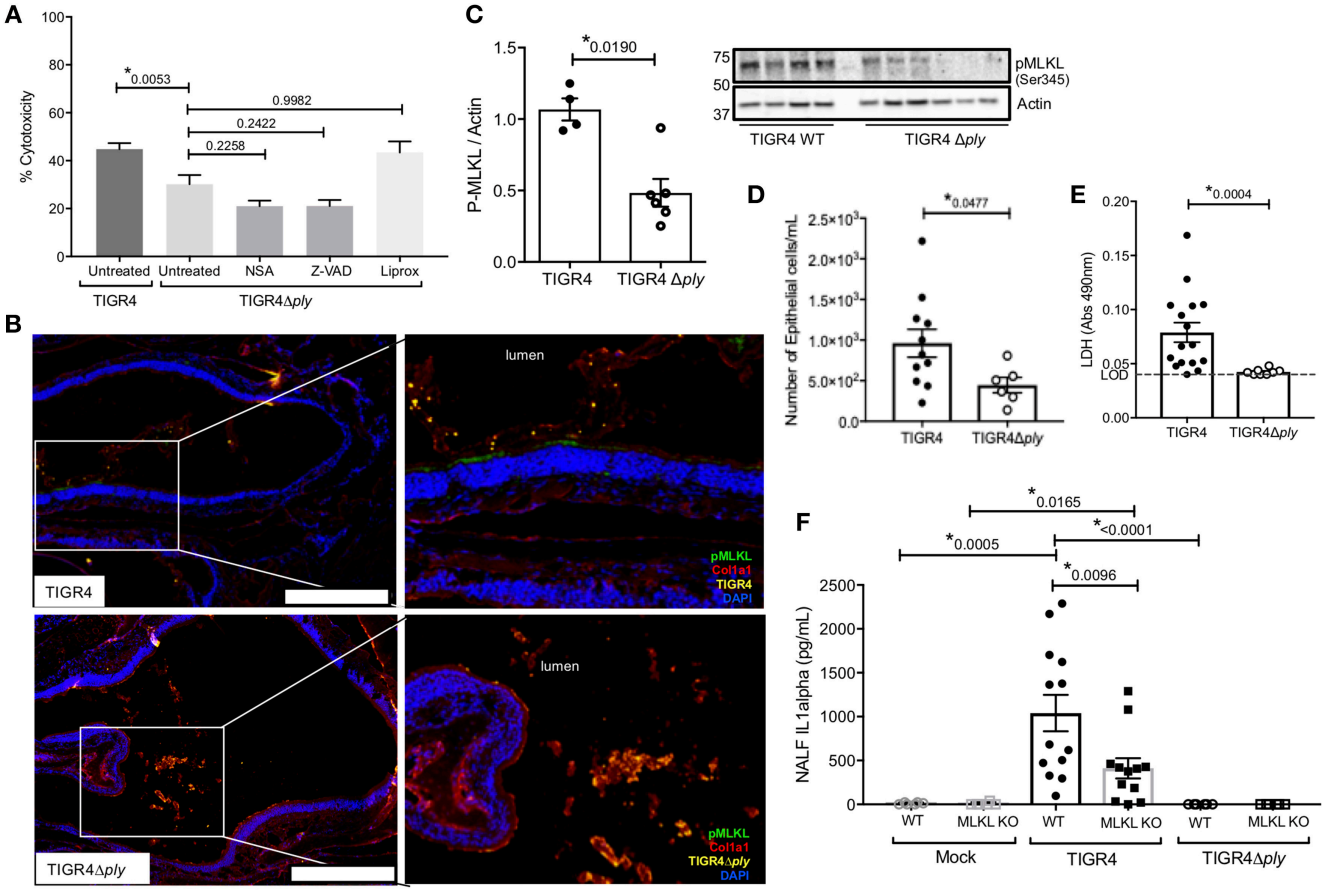
Necroptosis during *Spn* colonization is pneumolysin dependent. **(A)** FaDu pharyngeal cell death as measured by percent LDH release from cells pre-treated with normal media or media containing 10 μM of either the MLKL inhibitor Necrosulfonamide (NSA), the general caspase inhibitor ZVAD, or the ferroptosis inhibitor Liproxstatin-1 (Liprox), for 1 h then challenged overnight (15 h) with TIGR4 or TIGR4Δply at an MOI of 10. One-way analysis of variance (*F* = 11.86, *p* < 0.0001); *P*-values listed on graph; * indicates a value <0.05 (See also Figure S3B). **(B)** Representative immunofluorescent images of nasal turbinates from wildtype mice colonized with TIGR4 or TIGR4Δply, at day 7 post inoculation with 10^5^ CFU. Turbinates fixed and stained for collagen-la (red), Spn (yellow), pMLKL (green), and DAPI (Blue). Imaged at 40X magnification and Tile Scan assembled. White scale bars indicate 250 μm. **(C)** Western blot and densitometry for pMLKL and actin in nasal homogenates from mice colonized with TIGR4 or TIGR4Δply. Nasal lavage at 7-days post intra-nasal inoculation with TIGR4 or TIGR4Δply analyzed for **(D)** nEC sloughing, **(E)** lactate dehydrogenase (LDH), and **(F)** IL-1α. LDH Absorbance at 490 nm LOD normalized to absorbance of uninfected control NALF. Mann-Whitney *U*-test used for two-way comparisons and Kruskal-Wallis test with Dunn's post-test for multiple comparisons (Two infection experiments; total *n* = 10–14 animals per genotype). *P*-values listed on graph; * indicates a value < 0.05. (See also Figures S2, **S3** and Table S2).

Given our findings, we examined whether the observed nEC necroptosis was initiated by *Spn's* PFT. Infection of FaDu cells with Ply deficient *Spn* (TIGR4Δ*ply*) resulted in less cell death compared to infection with wildtype *Spn* (TIGR4); moreover, pre-treatment of cells with NSA, Z-VAD-fmk, or Liprox did not further reduce damage caused by the Ply-deficient *Spn* (Figure 3A). Unlike TIGR4 colonized mice, nasal sections and homogenates from WT mice colonized with TIGR4Δ*ply* did not show significant activation of MLKL (i.e., positive pMLKL staining) in *Spn*-associated regions (Figures 3B,C). Notably, nasal homogenates from these mice did not show meaningful amounts of cleaved caspase-3 (Figure S2B). NALF from WT mice colonized with TIGR4Δ*ply* were similar to that from MLKL KO mice colonized with TIGR4, with fewer sloughed nECs (Figure 3D) and drastically reduced levels of detectable LDH (Figure 3E), and IL-1α (Figure 3F) when compared to NALF from WT mice colonized with TIGR4. Collectively, these data suggest that the necroptosis during colonization by *Spn* is Ply-dependent.

### Necroptosis Alters the Innate Immune Response to Colonizing *Spn* and Decreases the Duration of Colonization

**Figure 4:**
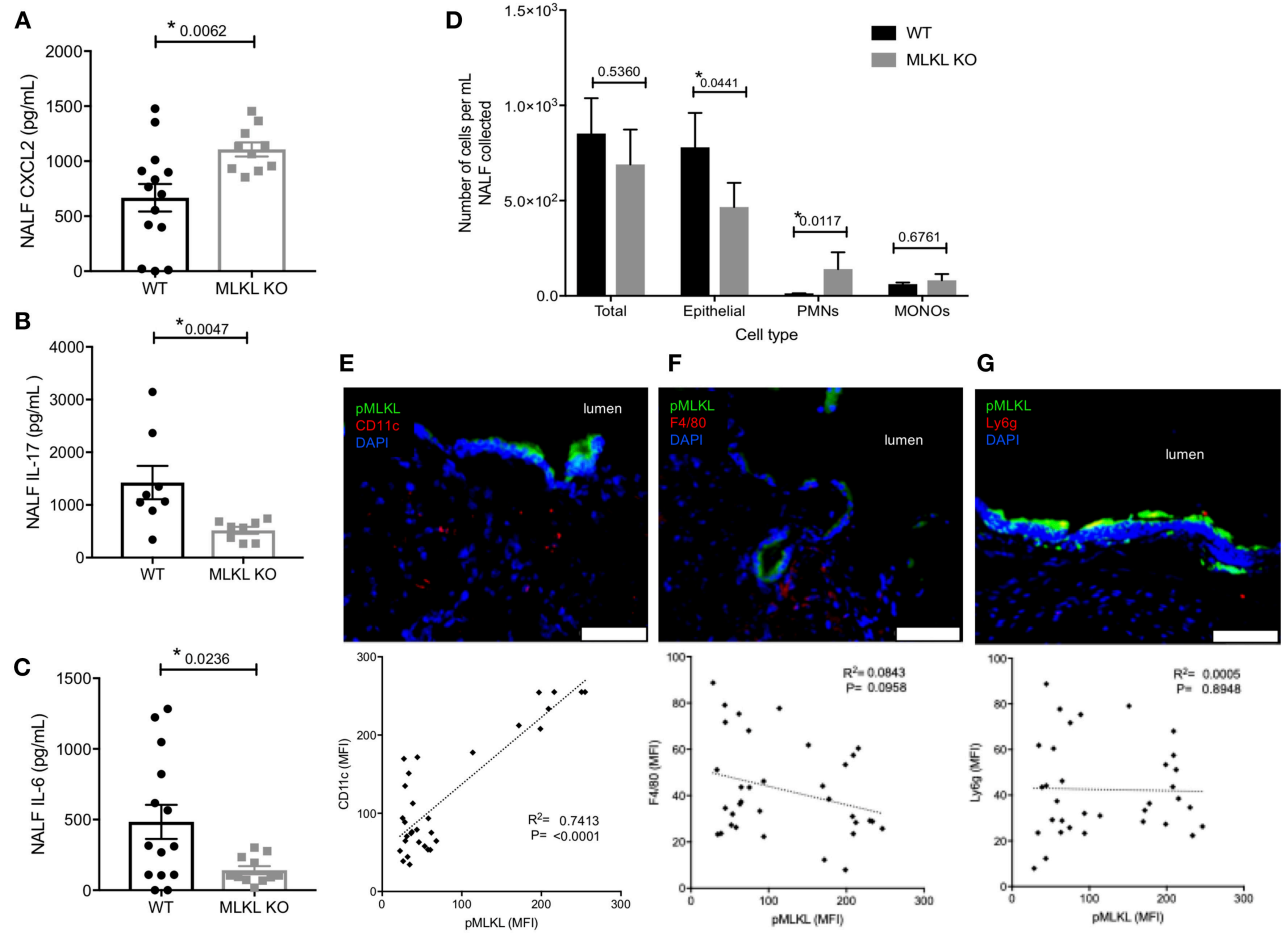
Localized PFT-induced necroptosis affects the innate immune response to colonizing *Spn*. Nasal lavage at day 7 post intra-nasal inoculation with 10^5^CFU TIGR4 analyzed for **(A)** CXCL2, **(B)** IL-17, and **(C)** IL-6. Mann-Whitney *U*-test used for comparisons. *n* = 10–14 animals per group; *P*-values listed on graph; * indicates value < 0.05. **(D)** Quantification of epithelial cells, polymorphonuclear cells (PMNs), and mononuclear cells (MONOs) in NALF at day 7 post intra-nasal inoculation with TIGR4 as quantified by HEMA stained Cytospins. One-way analysis of variance (*F* = 14.85, *p* = < 0.0001); *P*-values listed on graph; * indicates value < 0.05. **(E,F)** Representative immunofluorescently stained nasoturbinate section from wildtype mice colonized with TIGR4, at day 7 post inoculation, and correlation of pMLKL to cell-specific marker in stained sections. Sections stained for pMLKL (green), DAPI (blue), and **(E)** CD11c (*n* = 35, *R*^2^ = 0.7413, *p* < 0.0001), **(F)** F4/80 (*n* = 37, *R*^2^ = 0.08428, *p* = 0.0958), or **(G)** Ly6g (*n* = 35, *R*^2^ = 0.00056, *p* = 0.8948) in red. Imaged at 40X magnification and Tile Scan assembled. White scale bars indicate 50 μm (see also Table S2 for other cytokines tested but either unchanged or below limit of detection).

**Figure 5:**
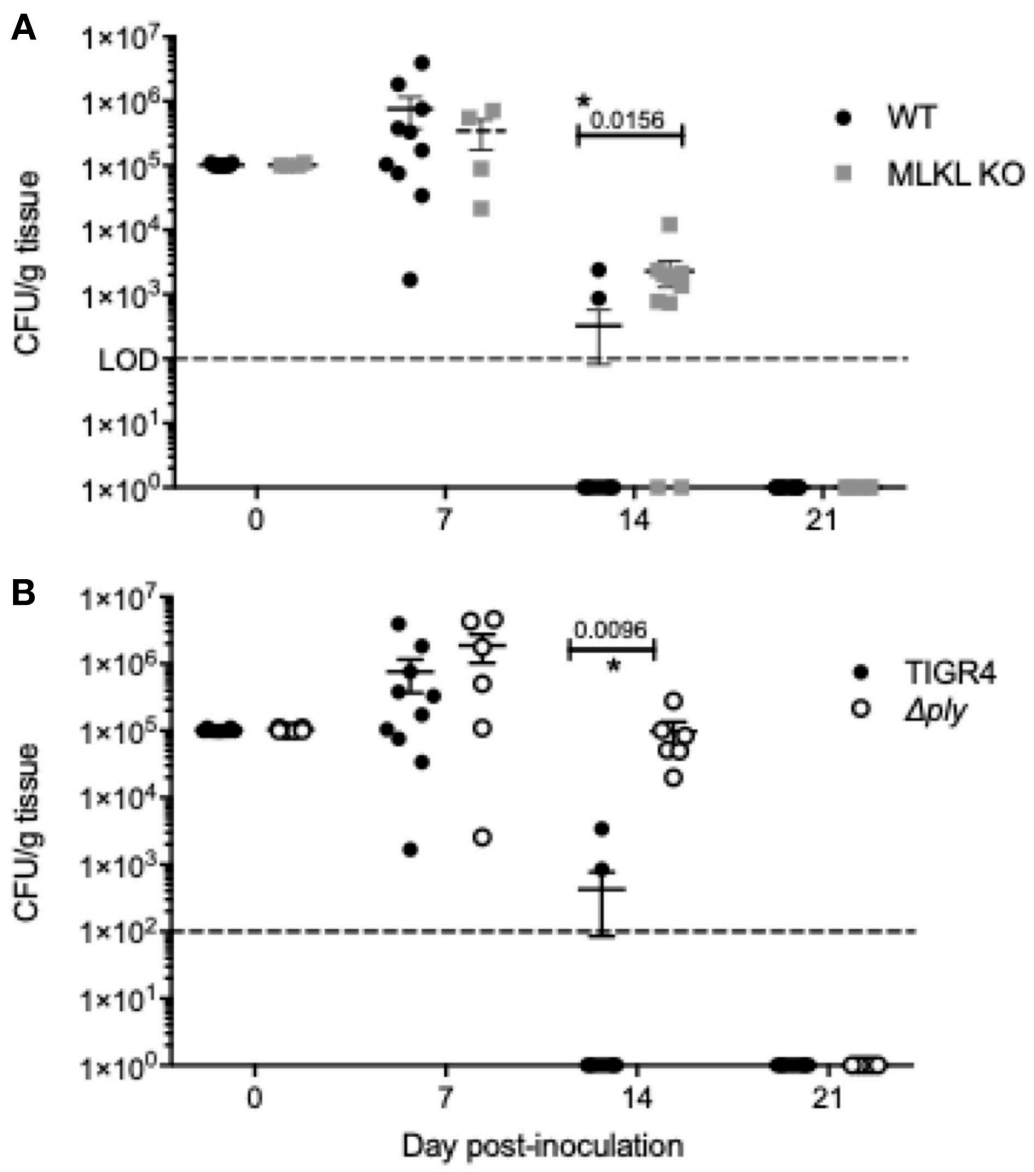
Inhibition of Ply-mediated necroptosis decreases the rate of *Spn* clearance. Number of *Spn* in nasal homogenates of **(A)** wildtype and MLKL KO mice colonized with TIGR4 and **(B)** wildtype mice colonized with TIGR4 or TIGR4Δply collected at days 0, 7, 14, and 21 post-inoculation. Mean ± SEM shown. *n* = 5 – 10 mice/group; two-way ANOVA with Sidak multiple comparisons, * = *P* < 0.05. Dashed line indicates limit of detection. Note that day 14 and 21 homogenates also plated in 100 μL spread plates to confirm clearance (limit of detection 100 CFU/g tissue).

We sought to better characterize how necroptosis influenced the innate immune response to pneumococcal colonization. NALF collected from MLKL KO mice at 7-days post-inoculation contained more of chemokine ligand-2 (CXCL2) (Figure 4A), a known neutrophil chemoattractant (45). NALF isolated from MLKL KO mice also had strikingly lower levels of the key colonization clearance cytokines IL-17 (Figure 4B) and IL-6 (Figure 4C). Despite having fewer sloughed nEC, TIGR4 colonized MLKL KO mice had more polymorphonuclear cells (PMNs) in their NALF (Figure 4D) 7-days post-inoculation than WT controls. In addition to the luminal cellular responses detected in NALF, we examined the submucosal immune cell population by fluorescent microscopy of nasal tissue sections. Fluorescent intensity for CD11c, an immune cell marker often associated with mature dendritic cells, positively correlated with that of pMLKL (*R*^2^ = 0.7413, *p* < 0.0001, *n* = 33) (Figure 4E) in nasal sections from WT colonized mice. Conversely, pMLKL-associated fluorescence did not correlate with that of F4/80, a common macrophage marker (*R*^2^ = 0.0843, *p* = 0.0958), or Ly6G, a common neutrophil marker (*R*^2^ = 0.0006, *p* = 0.8948) (Figures 4F,G). Importantly, when colonized with TIGR4, WT mice cleared pneumococcal colonization faster than the MLKL KO mice (Figure 5). Of note, differences in cytokine levels at day 7 were not due to differences in bacterial burden as these were equivalent at this time point. Thus, cell death by necroptosis during *Spn* colonization contributes toward greater amounts of pro-inflammatory cytokines previously associated with *Spn* clearance, reduced neutrophils, recruitment of potential antigen-presenting cells, and subsequently more rapid elimination of bacteria from the site of colonization.

### Nasopharyngeal Ply-Mediated Necroptosis Promotes Antibody Production Against *Spn* Surface Components

**Figure 6:**
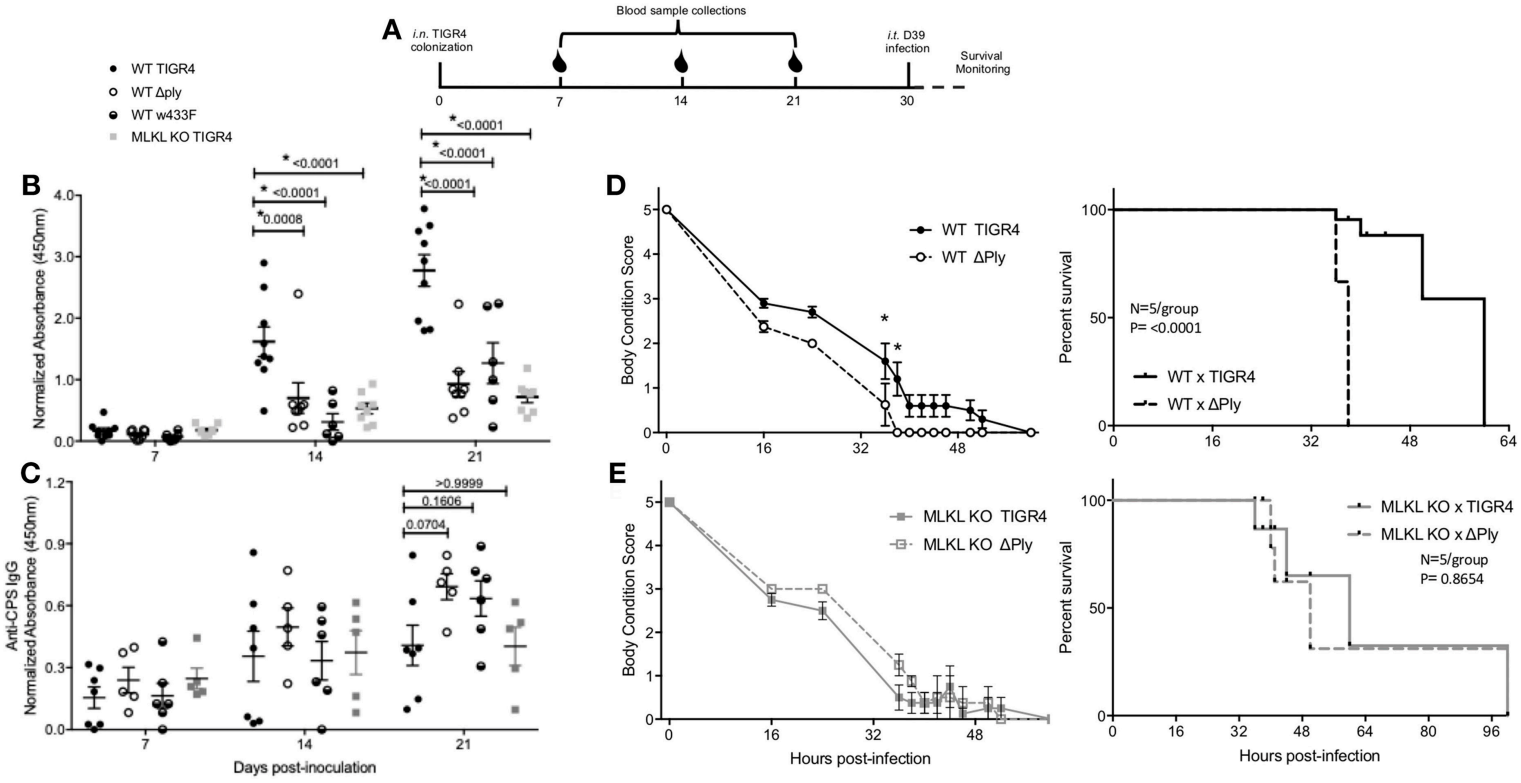
Necroptosis initiates protective, adaptive immunity against colonizing *Spn*. **(A)** Representative diagram of challenge and sample collection from mice in secondary lethal infection model. Serum IgG against **(B)** PspA or **(C)** type 4 capsular polysaccharide from wildtype (WT) or MLKL KO mice colonized with TIGR4, TIGR4Δply, or TIGR4w433F. Two-Way repeated measures ANOVA with Sidak's multiple comparisons test (Interaction *F* = 9.21, *p* = 0.4380; Time *F* = 59.31, *p* < 0.0001). *n* = 5–8 mice/group. **(D,E)** WT and MLKL KO mice colonized with wildtype TIGR4 (solid) or TIGR4Aply (dashed) and re-challenged intra-tracheally at day 30 post colonization inoculation with a lethal infectious dose of D39 (106). Body condition score and survival of **(D)** WT and **(E)** MLKL KO mice after intratracheal challenge. Body condition scores analyzed by Two-Way ANOVA with repeated measures (WT: Interaction *F* = 1.169, *p* = 0.1,030; Time *F* = 88.67, *p* < 0.0001) (KO: Interaction *F* = 0.8801, *p* = 0.3386; Time *F* = 90.45, *p* < 0.0001). Comparisons of time points with *p*-value < 0.05 indicated by asterisk. Survival analyzed by Mantel-Cox Log-rank test (WT: Chi square = 15.34, *p* < 0.0001) (KO: Chi square = 0.2603, *p* = 0.6099). *P*-values listed on graphs * indicates value < 0.05. (See also Figure S5). Mean ± SEM plotted for all panels.

Development of antibody against *Spn* surface proteins is a key facet of naturally acquired protective immunity (46). Given the delay in *Spn* clearance and reduced CD11c^+^ cell presence within the submucosa, we tested whether Ply-mediated necroptosis influenced the production of anti-*Spn* antibodies during colonization. To do this we measured the production of antibody against pneumococcal surface protein A (PspA). Briefly, PspA is a highly-conserved pneumococcal antigen, against which antibodies are protective (47). Serum from WT mice colonized with TIGR4 had more anti-PspA IgG than MLKL KO mice colonized with the same. Similarly, WT mice colonized with TIGR4 had higher anti-PspA IgG than WT mice colonized with TIGR4Δ*ply*. Along these lines, colonization of WT mice with an isogenic strain encoding the non-lytic Ply toxoid (TIGR4w433F) resulted in less anti-PspA IgG than WT mice colonized with TIGR4, implicating the necroptotic activity of the toxin as necessary for the immune-stimulatory effect of Ply (Figure 6B). Interestingly, these differences in antibody response were not observed in the T cell independent IgG response against type 4 capsular polysaccharide (Figure 6C), suggesting that necroptosis mediated inflammation in particular impacts antigen-presentation. The lack of an IgG response against PspA in colonized MLKL KO mice was not due to their inability to produce IgG or their ability to respond to mucosal protein challenge (Figure S4). Thus, Ply-mediated necroptosis positively influences the production of anti-pneumococcal antibody against T cell dependent antigens of colonizing *Spn*.

### Necroptosis Initiates the Development of Protective Immunity

Finally, we tested whether Ply-mediated necroptosis impacted naturally-acquired protective immunity, i.e., survival during subsequent lethal intra-tracheal challenge with a different serotype of *Spn*. To do this we used the following schema (Figure 6A); note that 21-days after colonization with TIGR4, no bacteria were detectable within nasal homogenates of mice (Figure 5). All mice, regardless of background died following secondary lethal D39 serotype 2 challenge. However, mice that had initially been colonized with TIGR4Δ*ply* succumbed more rapidly to infection vs. those previously colonized with TIGR4 (Figure 6D). Moreover, MLKL KO mice first colonized with TIGR4 were equivalent in susceptibility to D39 as MLKL KO mice colonized with TIGR4Δ*ply* (Figure 6E). This decreased mortality was not due to antibody against Ply, as shown by the increased susceptibility of WT mice colonized by TIGR4w433F and subsequently challenged with D39 (Figure S5). Note that a direct comparison between WT and MLKL KO mice was not made since we have already demonstrated that MLKL KO mice are protected against severe forms of *Spn* disease, such as the pneumonia modeled here in our secondary infection (15, 18). All together, these data demonstrate that PFT-mediated necroptosis during colonization is a key initiator of protective immunity against subsequent challenge by *Spn* of a different serotype.

## Discussion

The strikingly detrimental effects of necroptosis on tissue injury are well-documented (12, 48). This includes within the lower respiratory tract during infection with diverse PFT-producing bacterial pathogens (15, 16, 18). Yet, necroptosis also plays a pivotal role in the control of intracellular pathogens which inhibit apoptotic signaling. For example, necroptosis aborts the replication of viruses that have blocked caspase activation (14, 49, 50). In some instances, cell death by necroptosis may even serve to limit pathological inflammation through the divergence from other even more inflammatory forms of necrosis (51). Along such lines, and at the onset of our study, whether necroptosis was beneficial or detrimental in regards to infectious outcomes against colonizing pathobionts in the nasopharynx was unknown. Our results herein demonstrate that despite the associated tissue damage, cell death by necroptosis is beneficial and serves as a key initiator of innate and adaptive immunity against the colonizing PFT-producing pathogen, in this instance *Spn*. The impact of these findings is broad and may further extend to the numerous other PFT-producing mucosal colonizers.

Much of the damage resultant from necroptosis is due exacerbated inflammation. Molecules that contain DAMPs and other alarmins, such as IL-1α and IL-33, are strong immune stimulants which interact with nearby cells to induce proinflammatory cytokine signaling, initiate recruitment of antigen presenting cells (APC), and serve to activate APCs (52). Our observations that IL-1α, IL-33, and the key clearance cytokine IL-17 were increased in lavage from *Spn* colonized WT mice, but not MLKL KO mice or WT mice colonized with *Spn* lacking Ply, as well as the observed correlation of pMLKL with submucosal CD11c^+^ cells suggest that Ply-induced necroptosis may be the mechanism by which these key cytokines, which drive APC recruitment, are released during colonization. Notably, we did not detect IL-1β, suggesting that inflammasome activity was negligible and that pyroptosis is not a contributor to the release of these alarmins. In turn, the observed delay in *Spn* clearance seen in MLKL KO mice is most likely related to the reduction of these cytokines, as the clearance of *Spn* from the murine nasopharynx is accelerated by IL-1 family cytokines and IL-17 signaling (53–55). Notably, increased neutrophil infiltration has been reported for IL-1α KO mice (56). Thus, the increased levels of PMNs seen in NALF of MLKL KO mice is consistent with the decreased release of IL-1α and the greater levels of CXCL2 that were observed.

The role of PFTs, specifically Ply, during colonization and disease is multifaceted and not fully understood. For example, Ply is important in the initial establishment of *Spn* nasopharyngeal colonization (57); however this effect diminishes over time and mutants lacking Ply colonize for longer (58–60). Necroptosis may also increase *Spn* transmission as it has been shown that Ply-dependent inflammation is key in the transmission of *Spn* to a new host (36). Previous publications have shown that the Ply-induces cytokine and chemokine production which ultimately results in increased antigen delivery to lymphoid tissues and recruitment of APCs (59, 61). Our observations that MLKL KO mice and WT mice colonized with Ply mutant strains have reduced CD11c^+^ cell recruitment, diminished IgG response to *Spn* protein antigens, and delayed *Spn* clearance, is in agreement with these prior reports. Moreover, these data add the new understanding that programmed necrosis is a vital aspect of this process; i.e., facilitating the release of alarmins from nEC which help to activate the immune system. One consideration is that we did not perform flow cytometry to quantify distinct immune cell populations. While this was attempted, we were unable to isolate sufficient number of cells from the highly localized areas of *Spn* colonization to provide trustworthy results.

Given the latter, these results suggest the ability to undergo necroptosis is one way the host immune system is able to recognize and respond differently to potential pathogens (i.e., those producing PFTs) vs. non-threatening bacteria which colonize the same niche. Necroptosis-facilitated recruitment of APCs and the associated enhanced generation of protective antibody would in turn reduce the likelihood of severe disease caused by subsequent versions of the same PFT-producing pathogen. Extrapolating from this, our results further suggest that necroptosis induced during viral infection may also facilitate the recruitment of APCs and the generation of an adaptive immune response to viral antigens. Thus, further studies on whether directed necroptosis and resulting alterations in the local inflammatory signaling can be used to trigger adaptive immunity are worth pursuing. Open questions include the specific role of established and newly identified alarmins such as IL-1α and IL-33, in these processes.

A beneficial role for necroptosis during colonization is in stark contrast to that observed during severe bacterial diseases. Within the nasopharynx and during asymptomatic colonization, necroptosis serves to release factors that initiate a robust immune response and protect the host. These dual consequences reflect the double-edged sword which is the immune system. Further studies examining how the host utilizes necroptosis as an immune stimulus in response to asymptomatic viral infections, viral and bacterial co-infections, polymicrobial bacterial infections, and at other anatomical sites are warranted. In summary, our observations have important implications on our understanding of mucosal immunity and the co-evolution of the immune system with obligate mucosal pathogens like *Spn*.

## Ethics Statement

This study was carried out in accordance with the recommendations of the University of Alabama at Birmingham Institutional Animal Use and Care Committee (IACUC) in compliance with the federal regulations set forth in the Animal Welfare Act and the recommendations of the National Institutes of Health Guide for the Care and Use of Laboratory Animals. All procedures used in this study were approved by the IACUC under protocols #20479 and #21231.

## Author Contributions

AR and TB carried out the experiments. AR, TB, NG-J, and CO contributed to the design and conceptualization of the project. AR and CO wrote the manuscript. All authors provided critical commentary to help shape the manuscript.

### Conflict of Interest Statement

The authors declare that the research was conducted in the absence of any commercial or financial relationships that could be construed as a potential conflict of interest.
