# Supplementary material for: Necroptotic Cell Death Promotes Adaptive Immunity Against Colonizing Pneumococci
Source: Front Immunol. 2019 Apr 4;10:615. doi: 10.3389/fimmu.2019.00615 (PMC6459137; doi:10.3389/fimmu.2019.00615)
Supplement: Supplementary file 1 [file Data_Sheet_1.PDF]

Necroptotic Cell Death Promotes Adaptive Immunity Against Colonizing Pneumococci

Authors: A. N. Riegler,[1] T. Brissac,[1] N. Gonzalez-Juarbe[1,2], C. J. Orihuela[1*]

Supplemental Materials:

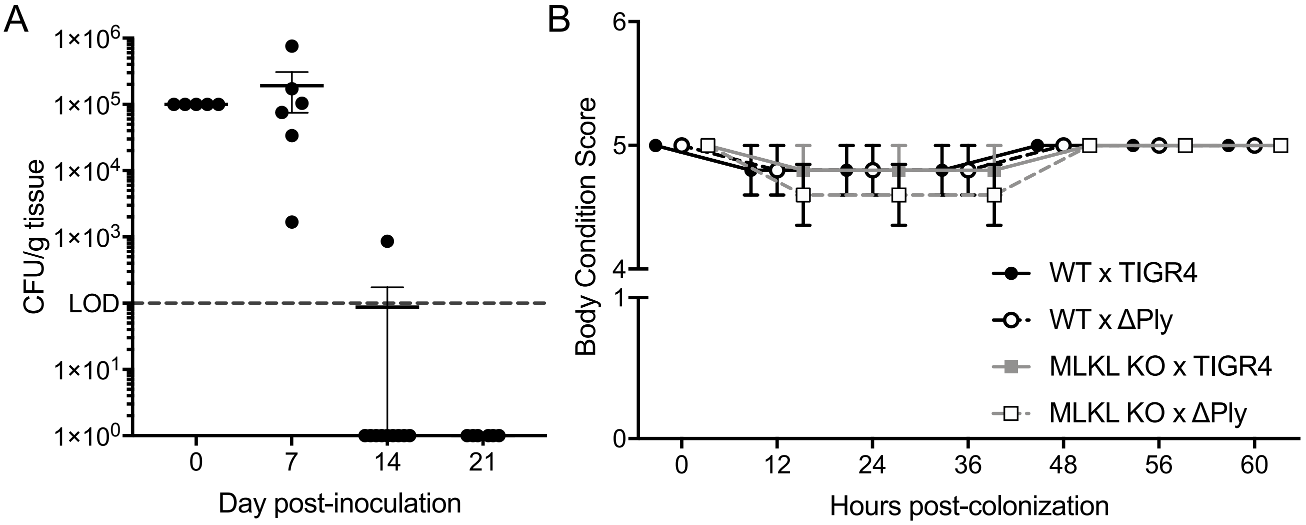

C

Score	Assessment	**Description** (meets any of the following descriptions)
5	No Detectable Pain or Distress; HEALTHY	Normal, well groomed, alert, active, calm, normal apetite
4	Minimal Natural Discomfort but HEALTHY	Well groomed, alert, and active but observable signs of natural distress such as hair loss, not as active as other cage mates, less responsive to stimuli.
3	Minimal Discomfort or Distress but HEALTHY	Alert and active with minimal observable signs of distress. Not well groomed, less activity than cage mates, less responsive to stimuli, overstimulated/overweight, awkward gait or observable joint distress (red joints).
2	Mild Pain and Distress; SICK	Not well groomed, awkward gait, slightly hunched, perforin staining on eyes/face.
2-	Moderate Pain and Distress; SICK	Rough hair coat, squinted eyes, moves slowly, walks hunched and/or slowly, slight dehydration (poor skin turgor), pruritic (frequentor long scratching/itching reflex; >2/10min or lasting >30sec), not eating or drinking, “rolling” reflex
1	Severe Pain and Distress; MORIBUND	Very rough hair coat, severe dehydration (sunken eyes, lack of skin turgor), non-responsive when coaxed, slow but detectable breath rate (<55 breaths/min).
0	Dead	No detectable breathing, movement, or pain response (toe pinch) for >30sec.

Figure S1. Nasopharyngeal colonization model. (A) Nasopharyngeal burden from nasal homogenates at 7-days post-inoculation. Mean ± SEM plotted. (B) Body condition scores of colonized WT and MLKL KO mice for the initial 60 hours’ post-inoculation. Mean ± SEM shown. Note that all colonized mice remained at a healthy score of 4-5 for the duration of colonization. (C) Table defining body condition score assessment criteria.

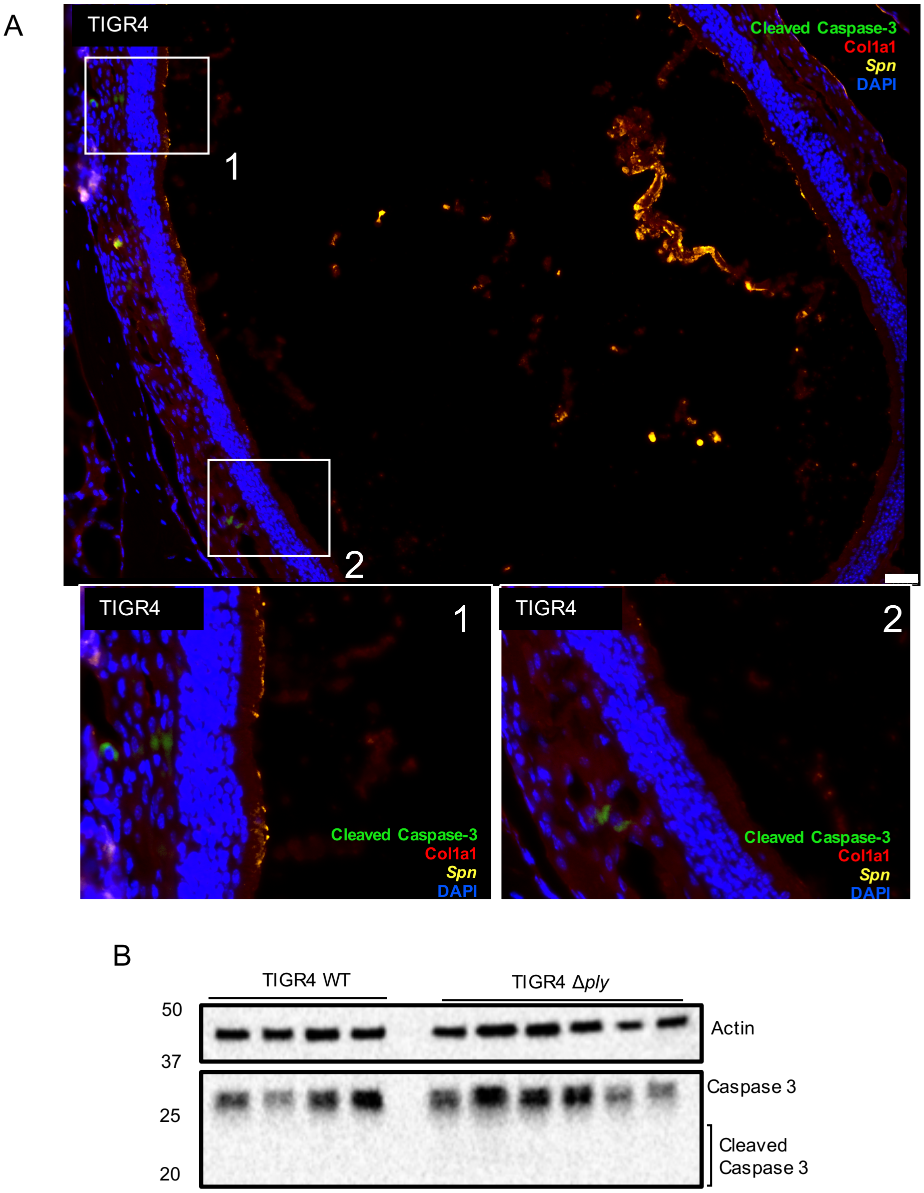

Figure S2. Amount of apoptosis during *Spn* colonization is negligible. (A) Nasoturbinate from TIGR4 colonized WT mouse immunofluorescently stained for cleaved caspase-3 (green), collagen1a (red), *Spn* (yellow), and DAPI (blue). Panels 1 and 2 indicating the limited cell number staining positive for active caspase-3. Notably, most sections showed no active caspase-3. Scale bar indicates 25µm. (B) Western blot for cytoskeletal actin and pro- and cleaved caspase-3 in nasal homogenates from TIGR4 and TIGR4Δ*ply* colonized mice.

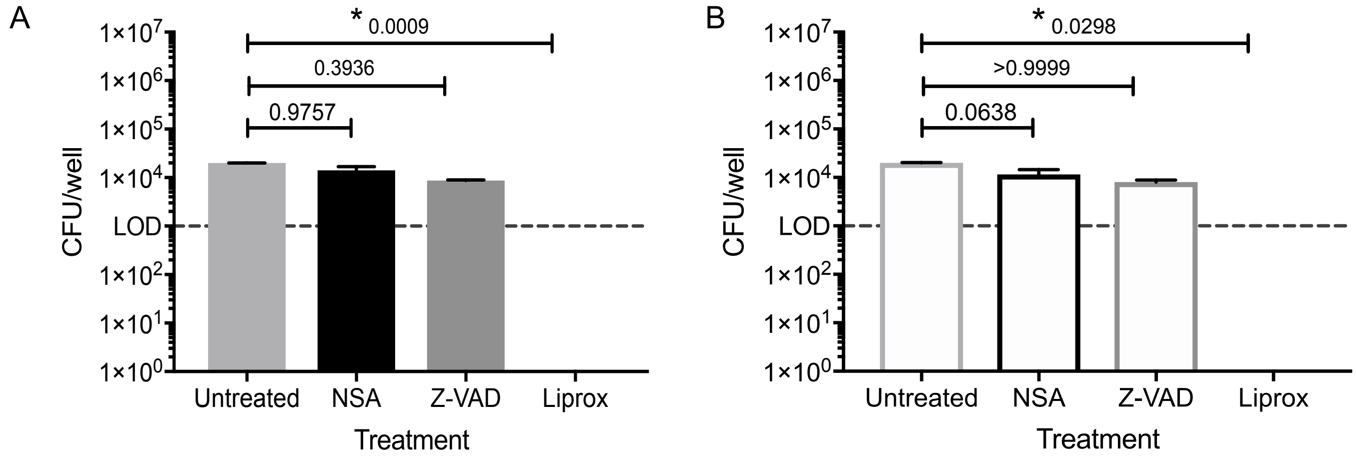

Figure S3. Reduction in FaDu cytotoxicity is not due to *Spn* growth differences during treatment. Infectious burden (CFU/well) following 1 hour inhibitor pre-treatment and overnight (15h) challenge with (A) TIGR4 or (B) TIGR4Δ*ply*. Dashed line indicates limit of detection. One-way analysis of variance used for comparisons and individual p-values shown on graph. Mean ± SEM plotted.

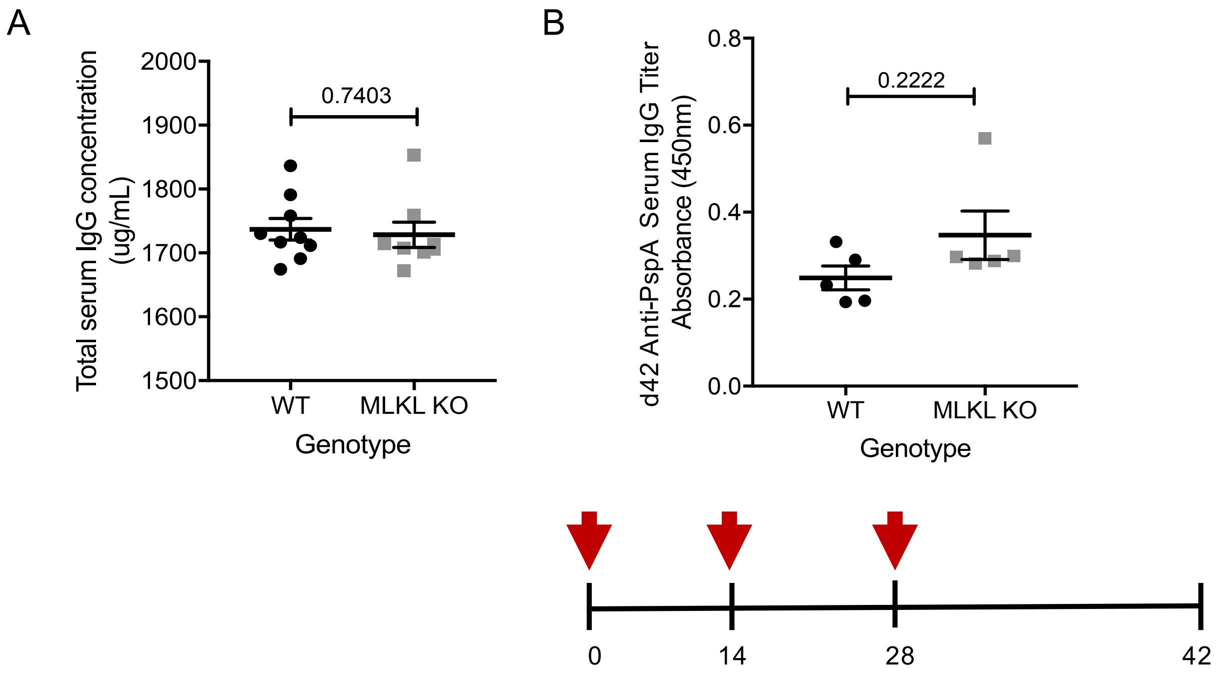

Figure S4. Reduction in antibody in MLKL KO mice is not a genotype defect. Total serum IgG at day 21 post inoculation of WT and MLKL KO mice colonized with TIGR4 (n=8-9 mice per genotype). (B) Day 42 serum anti-PspA IgG of mice given 5µg rPspA intranasally at days 0, 14, and 28. Mann-Whitney U test for comparison (n= 5 mice per genotype). Mean ± SEM shown.

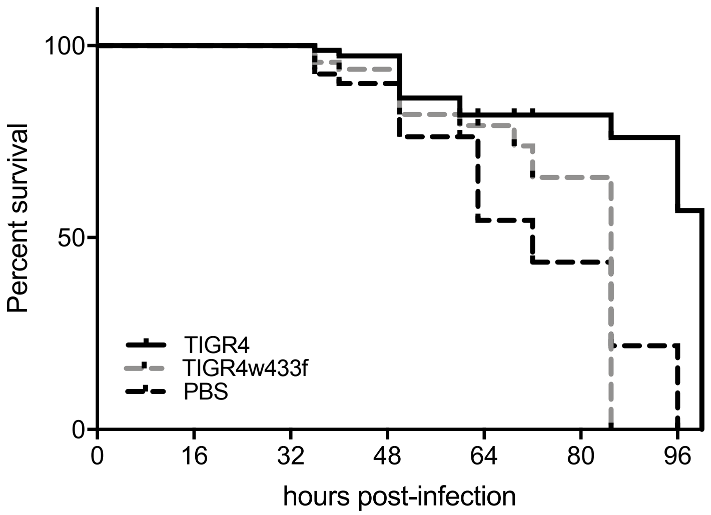

Figure S5. Protection against secondary lethal challenge is not due to anti-pneumolysin antibody. Survival of WT mice, initially colonized with wildtype TIGR4 (solid), TIGR4w433f (grey dashed), or mock colonized (black dashed), and re-challenged intra-tracheally at day 30 post colonization-inoculation with a lethal infectious dose of D39 (10^6). Mantel-Cox Comparison, N=16 mice/group, p=0.0026.

Table S1. Reagents and Resources. Details for all key resources used for data collection.

REAGENT or RESOURCE	SOURCE	Details
**Antibodies**		
Mouse Anti-Collagen I Antibody	Abcam	Cat#ab90395
Rabbit anti-mouse pMLKL Antibody	Abcam	Cat#ab196436
Rabbit anti-Human, Mouse, Rat, Monkey Caspase-3 Antibody (western)	Cell Signaling Technologies	Cat#9662S
Rabbit anti-Mouse pMLKL Antibody (western)	Cell Signaling Technologies	Cat#37333S
Rabbit anti-Mouse MLKL Antibody (western)	Cell Signaling Technologies	Cat#37705
Rabbit anti-Human MLKL Antibody (western)	Abcam	Cat#ab184718
Rabbit anti-Human pMLKL Antibody (western)	Cell Signaling Technologies	Cat#91689S
Rabbit anti-Cytoskeletal Actin Antibody (western)	Bethyl	Cat#A300-485A
Affinipure Goat Anti-Rabbit IgG (Peroxidase)	Jackson Immuno	Cat#111-035-144
Rabbit Anti-Human, Mouse Active Caspase-3 Antibody	R&D Systems	Cat#AF835SP
Rabbit anti-serotype 4 Antibody	Statens Serum Institut	Cat#16747
Donkey anti-rabbit IgG (Dylight® 550)	Abcam	Cat#ab96920
Goat anti-rabbit IgG (FITC)	Jackson Immuno	Cat#111-095-003
Donkey anti-rabbit IgG (Rhodamine)	Jackson Immuno	Cat#711-025-152
Hamster anti-mouse CD11c (ALEXA Fluor® 647)	Biolegend	Cat#117314
Rat anti-mouse F4/80 (ALEXA Fluor® 647)	Biolegend	Cat#123121
Rat anti-mouse Ly-6G (ALEXA Fluor® 594)	Biolegend	Cat#127636
Rabbit anti-human/mouse active caspase-3	R&D Systems	Cat# AF835SP
Affinipure Goat Anti-mouse IgG	Jackson Immuno	Cat#115-000-003
Affinipure Goat Anti-mouse IgM	Jackson Immuno	Cat#115-005-003
Affinipure Goat Anti-mouse IgG (Peroxidase)	Jackson Immuno	Cat#115-035-003
Affinipure Goat Anti-mouse IgM (Peroxidase)	Jackson Immuno	Cat#115-035-020
**Bacterial Strains**		
Streptococcus pneumoniae: type 4 serotype strain TIGR4	ATCC	ATCC BAA-334
Streptococcus pneumoniae: type 4 serotype strain TIGR4Δ*ply*	Lizcano et al., 2010	N/A
Streptococcus pneumoniae: type 4 serotype strain TIGR4ply_{W433F}	Zafar et al., 2016	N/A

Streptococcus pneumoniae: type 2 serotype strain D39	NCTC	NTCT 7466
**Chemicals, Peptides, and Recombinant Proteins**		
NucBlue Live ReadProbes™ Reagent	Invitrogen	Cat#R37605
Recombinant pneumococcal surface protein A, rPspA	Ren et al., 2003	N/A
Recombinant pneumolysin, rPly	Douce et al., 2010	N/A
Necrosulfonamide	Tocris	Cat#5025
Z-VAD-fmk	R&D Systems	Cat#FMK001
Liproxstatin-1	Sigma-Aldrich	CAS#950455-15-9
HALT™ Protease and Phosphatase Inhibitor Cocktail	Thermo Scientific	Cat#78446
PROTOCOL HEMA 3 Manual Staining System and Stat Pack	Fisher HealthCare	Cat#22-122911
**Critical Commercial Assays**		
Pierce LDH Cytotoxicity Assay Kit	Thermo Scientific	Cat#88954
Mouse IL-1 Alpha/IL-1F1 DuoSet ELISA Kit	R&D Systems	Cat#DY400
Mouse/Rat IL-33 Quantikine ELISA Kit	R&D Systems	Cat#M3300
Mouse CXCL2/MIP-2 Duoset ELISA Kit	R&D Systems	Cat#DY452-05
Mouse IL-6 DuoSet ELISA Kit	R&D Systems	Cat#DY406-05
Mouse IL-17 DuoSet ELISA Kit	R&D Systems	Cat#DY413-05
Bicinchoninic Acid (BCA) Kit for Protein Determination	Sigma-Aldrich	Cat#BCA1
Clarity™ Western ECL Substrate	BIO-RAD	Cat#1705061
APEX™ Alexa Fluor® 488 Antibody Labeling Kit	Molecular Probes	Cat#A10468
Mouse CXCL1/KC DuoSet ELISA Kit	R&D Systems	Cat#DY453
Mouse IL-1β ELISA Set	BD Biosciences	Cat#559603
Mouse IL-7 DuoSet ELISA Kit	R&D Systems	Cat#DY407
Mouse IL-12 DuoSet ELISA Kit	R&D Systems	Cat#DY419
Mouse IL-13 DuoSet ELISA Kit	R&D Systems	Cat#DY413-05
Mouse IFNγ DuoSet ELISA Kit	R&D Systems	Cat#DY485
Mouse TNFα DuoSet ELISA Kit	R&D Systems	Cat#DY410
RDO Decalcification Solution	Electron Microscopy Sciences	Cat#6414301
**Mouse and Cell Lines**		
Human: FaDu cells	ATCC	HTB-43
Experimental Models: Organisms/Strains		
Mouse: C57BL/6	Jackson Laboratory	JAX#0006644
Mouse: $Mlkl^{tm1.2Wsa}/Mlkl^{tm1.2Wsa}$	Murphy et al., 2013	RRID#5614466
**Recombinant DNA and Protiens**		
pUAB099; rPspA expression	Ren et al., 2003	N/A
pET33(+) rPly; rPly expression	Douce et al., 2010	N/A

Software and Algorithms		
GraphPad Prism 8	GraphPad	https://www.graphpad.com/scientific-software/prism/
Leica Application Suite X; imaging	Leica Microsystems	https://www.leica-microsystems.com/products/microscope-software/details/product/leica-las-x-ls/

Table S2. NALF cytokines unchanged or below limit of detection.

Cytokine	C57<br>TIGR4 vs TIGR4Δ*ply*	C57 vs MLKL KO<br>TIGR4
CXCL1/KC	No significant differences.<br>P=0.6900, N=5-8 mice/group (TIGR4: Mean = 1.144 pg/ml, SEM= 0.5163; Δply: Mean= 1.698 pg/ml, SEM=0.7737)	No significant differences.<br>P=0.3440, N=5-8 mice/group (WT: Mean = 1.144 pg/ml, SEM= 0.5163; KO: Mean = 3.109pg/ml, SEM= 1.376)
IL-1β	All samples below limit of detection (31.3 pg/mL)	All samples below limit of detection (31.3 pg/mL)
IL-7	All samples below limit of detection (31.3 pg/mL)	All samples below limit of detection (31.3 pg/mL)
IL-12	All samples below limit of detection (39.1 pg/mL)	All samples below limit of detection (39.1 pg/mL)
IL-13	All samples below limit of detection (62.5 pg/mL)	All samples below limit of detection (62.5 pg/mL)
IFNγ	No significant differences.<br>P=0.969 , N=5-8 mice/group (TIGR4: Mean = 26.78 pg/ml, SEM= 0.1033; Δply: Mean= 26.52 pg/ml, SEM=0.1583)	No significant differences.<br>P=0.8185, N=5-8 mice/group (WT: Mean = 26.78 pg/ml, SEM= 0.1033; KO: Mean = 27.19 pg/ml, SEM= 0.1894)
TNFα	All samples below limit of detection (31.3 pg/mL)	All samples below limit of detection (31.3 pg/mL)

NALF collected at day 7 post-inoculation and examined by ELISA. Limit of detection listed for each ELISA with levels below detection limit. Means and SEM given for each group with detectable cytokine levels.
